# Incorporating neck circumference or neck-to-height ratio into the GOAL questionnaire to better detect and describe obstructive sleep apnea with application to clinical decisions

**DOI:** 10.3389/fnins.2022.1014948

**Published:** 2022-10-10

**Authors:** Ying Zhao, Xiangru Yan, Chunguang Liang, Liying Wang, Hui Zhang, Haitao Yu

**Affiliations:** Department of Nursing, Jinzhou Medical University, Jinzhou, China

**Keywords:** obstructive sleep apnea, GOAL questionnaire, neck circumference, neck-to-height ratio, screening diagnosis

## Abstract

**Objective:**

Although neck circumference (NC) and neck-to-height ratio (NHR) have been recognized as effective predictors of the clinical diagnosis of adult obstructive sleep apnea (OSA), they have not been included in the widely used GOAL questionnaire. Not coincidentally, the NHR has not been adequately considered in the development and validation of the STOP-Bang questionnaire, No-Apnea score and the NoSAS score. The motivation for the study was (1) to combine the GOAL questionnaire with the NC and NHR, respectively, to evaluate its predictive performance and (2) to compare it with the STOP-Bang questionnaire, the No-Apnea score, the NOSAS score, and the GOAL questionnaire.

**Materials and methods:**

This retrospectively allocated cross-sectional study was conducted from November 2017 to March 2022 in adults who underwent nocturnal polysomnography (PSG) or home sleep apnea testing (HSAT). In this paper, the GOAL questionnaire was combined with the NC and NHR, respectively, using logistic regression. The performance of the six screening tools was assessed by discriminatory ability [area under the curve (AUC) obtained from receiver operating characteristic (ROC) curves] and a 2 × 2 league table [including sensitivity, specificity, positive predictive value (PPV), negative predictive value (NPV), positive likelihood ratio (LR+), and negative likelihood ratio (LR−)] and compared under AHI ≥5/h, AHI ≥15/h, and AHI ≥30/h conditions.

**Results:**

A total of 288 patients were enrolled in the study. For all severity OSA levels, the sensitivity of GOAL+NC ranged from 70.12 to 70.80%, and specificity ranged from 86.49 to 76.16%. The sensitivity of GOAL+NHR ranged from 73.31 to 81.75%, while specificity ranged from 83.78 to 70.86%. As for area under the curve (AUC) value under ROC curve, when AHI ≥5/h, compared with GOAL (0.806), No-Apnea (0.823), NoSAS (0.817), and GOAL+NC (0.815), GOAL+NHR (0.831) obtained the highest AUC value, but lower than STOP-Bang (0.837).

**Conclusion:**

The predictive power of incorporating NC or NHR into the GOAL questionnaire was significantly better than that of the GOAL itself. Furthermore, GOAL+NHR was superior to GOAL+NC in predicting OSA severity and better than the No-Apnea score and the NoSAS score.

## Introduction

Obstructive sleep apnea (OSA) is a common form of sleep apnea disorder characterized by a decrease in pharyngeal muscle tone during sleep resulting in pharyngeal collapse, causing repeated partial or complete obstruction of the upper airway (Remmers et al., 1978). The physiological alterations are repeated narrowing and closure of the upper airway during sleep, resulting in a brief respiratory arrest of at least 10 s or a significant reduction in tidal volume, which in turn causes intermittent hypoxia and sympathetic activation with frequent microarousals (Nadeem et al., 2013). OSA is a common disorder with a prevalence of 9–38% in the general population (Senaratna et al., 2017; Lal et al., 2021). Snoring, apnea, fatigue, frequent awakenings, nocturia, and daytime sleepiness are all common symptoms of OSA (Chung et al., 2008). Risk factors for OSA include age, male gender, obesity, neck circumference (NC), abnormal craniofacial features, and female menopause (Young et al., 2004). Early diagnosis of OSA is crucial and untreated OSA may lead to the development of hypertension, cardiovascular disease, type 2 diabetes and neurocognitive dysfunction (Almendros et al., 2019; Tokunou and Ando, 2020; Ekin et al., 2021; Heilbrunn et al., 2021; Yeghiazarians et al., 2021).

The most accurate methods of diagnosing OSA are the use of polysomnography (PSG) in the laboratory or the employment of a home sleep apnea tester in the home (Kapur et al., 2017). However, these methods are time consuming, expensive, require specialist technician training (Chung et al., 2008) and are not readily available, especially in areas with limited economic resources. Globally, sleep testing centers have a large number of patients with suspected OSA waiting to be tested (Duarte et al., 2022). In this context, various OSA screening tools have emerged, and they are often developed based on the clinical risk factors, demographic, and anthropometric factors that constitute the main components of the disorder (Duarte et al., 2021). Examples include the GOAL questionnaire, the STOP-Bang questionnaire, the No-Apnea score, the NoSAS score, anthropometric indicators (neck circumference, neck-to-height ratio, etc.) (Liu et al., 2022). It provides great convenience for screening OSA patients.

According to statistics, abnormal pharynx and obesity play a very special role in the pathogenesis of sleep apnea, and neck fat may affect the pharyngeal characteristics (Gasa et al., 2019; Zhang et al., 2022). Various techniques and tools exist for the measurement of body fat, such as the determination of fat or water content by chemical dilution, 4K potassium isotope measurements, bioelectrical impedance and body density measurements (Hoffstein and Mateika, 1992). However, these techniques face the difficulty of large coefficient of difficulty and not easy to obtain in clinical screening work. Based on this dilemma, a number of alternative anthropometric measures have been chosen to represent the distribution of fat. Body mass index (BMI), a measure of body fat distribution, is associated with the severity of OSA disease (Topirceanu et al., 2020). However, BMI reflects overall fat distribution and does not adequately take into account neck fat distribution, which has limitations. Neck fat distribution has been inferred from NC, and some studies have shown that neck fat is thicker in OSA than in non-apnea snorers (Morinigo et al., 2022). Neck-to-height ratio (NHR) is a better indicator of body shape in a population than NC, and NHR has been suggested as a measure of neck obesity (Hoffstein and Mateika, 1992).

The GOAL is a newly developed and validated screening questionnaire that contains both objective and subjective variables and provides yes-or-no dichotomous answer. The GOAL questionnaire includes gender, obesity, age, and loud snoring with items scored from 0 to 4. A score of ≥2 indicates a higher risk of having OSA. The GOAL is the only questionnaire that does not include NC as a variable and has similar predictive performance compared to other validated screening tools (STOP-Bang questionnaire, NoSAS score, and No-Apnea score). So how would the predictive performance of the GOAL questionnaire change when combined with NC or NHR?

In light of the above, our study design had two main objectives: (1) to assess the predictive performance of the GOAL questionnaire in combination with the NC and NHR, respectively, in patients with OSA and (2) to compare it with the STOP-Bang questionnaire, the No-Apnea score, the NoSAS score, and the GOAL questionnaire, all of which are well-validated screening tools for OSA.

## Materials and methods

### Study subjects

This retrospective cross-sectional study evaluated patients attending the Sleep Monitoring Centre at the First Hospital of Jinzhou Medical University, Jinzhou City, Liaoning Province, between November 2017 and March 2022. Participants who were suspected of having a sleep breathing disorder by their treating physician were referred for assessment with nocturnal PSG or home sleep apnea test (HSAT). Participants were included in the following criteria: Aged 18 or above (including 18), suspected of having OSA, and total sleep duration ≥4 h. Exclusion criteria: patients with previously diagnosed OSA, patients previously treated with continuous positive airway pressure ventilation (CPAP), patients with missing anthropometric data and patients with incomplete answers on the scale. In addition, it should be noted that patients with neck disorders (such as thyroid hypertrophy, tonsil hypertrophy, skeletal, or neuromuscular disorders, etc.) were excluded in order to reduce unnecessary interference in the rational use of NC and NHR indicators. The study was approved by the center’s ethics committee before it began (registration number: LLSC2020008).

### Basic data collection

In this study, we collected general information such as patients’ name, age, gender, height, weight, neck circumference, hypertension, and assessed BMI and NHR. The Stop-Bang and NoSAS scales were filled out by patients and their families, and items in the scales were verified by a sleep technician to ensure their reliability The GOAL questionnaire and No-Apnea score were refined based on general information.

### Obstructive sleep apnea screening questionnaire

GOAL questionnaire (Duarte et al., 2020) contains four clinical parameters: male gender, obesity (BMI ≥30 kg/m^2^), age ≥50 years, and loud snoring. Each parameter contains a yes-or-no dichotomous answer (each positive answer is scored as 1), with a final score of 0–4. A score of ≥2 indicates a higher risk of OSA.

STOP-Bang questionnaire (Chung et al., 2008) contains 8 clinical parameters: snoring, fatigue, observed apnea, hypertension, BMI >35 kg/m^2^, age >50 years, NC >40 cm, and male gender. Each parameter contains a yes-or-no dichotomous answer (each positive answer is scored as 1). STOP-Bang questionnaire employments score of 3 to identify subjects at risk of OSA.

NoSAS score (Marti-Soler et al., 2016) is: 4 points for NC >40 cm, 3 points for 25 kg/m^2^ < BMI <30 kg/m^2^ or 5 points for BMI ≥30 kg/m^2^, 2 points for snoring, 4 points for over 55 years old, and 2 points for male gender. NoSAS scores range from 0 to 17. NoSAS score ≥8 is considered to be high risk for OSA.

No-Apnea score (Duarte et al., 2018) contains two objective parameters: NC and age. NC range is 1 point in 37.0–39.9, 3 points in 40.0–42.9 and 6 points in ≥43.0. The age range of 35–44 is 1 point, 45–54 is 2 points, and ≥55 is 3 points. The No-Apnea score ranges from 0 to 9, and ≥3 is considered positive.

### Obstructive sleep apnea diagnosis and severity

To assess the presence and severity of OSA, patients underwent a complete nocturnal PSG or home sleep apnea test (HSAT). Measurements included nasal airflow, chest and abdominal movement, pulse oximetry, snoring, and body position sensors. We used PSG to record synchronously for at least 7 h continuously. PSG/HSAT data were automatically analyzed, manually reviewed and corrected by sleep specialists, and finally interpreted and analyzed by sleep physicians according to the latest American Medical Association 2012 standard report (Berry et al., 2012). According to the American College of Physicians 2017 Guidelines for the Interpretation of Sleep and Related Events (Berry et al., 2017), apnea is defined as a 90% reduction in airflow that lasts for at least 10 s. Hypopnea was defined as a 30% reduction in airflow lasting at least 10 s and a 3% decrease in oxygen saturation The apnea-hypopnea index (AHI)/respiratory event index (REI) was calculated based on the average of apnea and hypopnea events per hour of sleep (Malhotra et al., 2021).

Polysomnography diagnosed AHI and HSAT diagnosed REI classified patients as having no apnea (AHI/REI, <5), mild OSA (AHI/REI, 5–14), moderate OSA (AHI/REI, 15–29), and severe OSA (AHI/REI, ≥30). Participants’ No-Apnea scores, NoSAS scores, STOP-Bang scores, GOAL scores, GOAL+NC, and GOAL+NHR were compared to their PSG or HSAT obtained AHI or REI results, respectively.

### Statistical analysis

SPSS 26.0 statistical software was used for analysis. Results were summarized by frequency and percentage (categorical variables), median and interquartile range (IQR) (continuous variables). Categorical variables were compared using the chi-square test, while continuous variables were assessed using the non-parametric Mann–Whitney test. Sensitivity, specificity, PPV, NPV, LR+, and LR− were calculated using a 2 × 2 column table and reported at their respective 95% confidence intervals (CI). Using logistic regression in Medcalc 20.0 software, the GOAL questionnaire was combined with NC and NHR, respectively and AUC values were calculated for GOAL+NHR and GOAL+NC, respectively. The discriminatory ability of GOAL+NHR, GOAL+NC, STOP-Bang, No-Apnea, NoSAS and GOAL in screening for any severe level of OSA was judged by the AUC obtained from the receiver operating characteristic (ROC) curves. AUC >0.7 was considered clinically significant [1]. All prediction parameters were calculated at three generally accepted AHI thresholds: 5.0 ≥ /h, 15.0 ≥ /h, and 30.0 ≥ /h. Statistical significance level was set as *P* < 0.05.

## Results

Based on our inclusion and exclusion criteria, 288 OSA patients were collected at the sleep monitoring center and assigned to 2 independent data sets; The normal group (*n* = 37; 12.8%) or OSA group (*n* = 251; 87.2%). According to Table 1, the median age of the 288 patients was 46 years (IQR 35–54 years), height of 170 cm (IQR 165–175 cm), weight of 81 kg (IQR 70–90 kg), BMI of 27.74 kg/m^2^ (IQR 24.99–30.78 kg/m^2^), NC of 41 cm (IQR 38–43 cm), NHR of 0.24 (IQR 0.22–0.25), The median values of No-Apnea, NOSAS, Stop-Bang, GOAL, AHI, and lowest SpO_2_ were 5 (2–7) points, 11 (7–13) points, 5 (3–6) points, 27.4 (11.2–65.4) times/h, and 83% (74–87), respectively.

**TABLE 1:** General information on the study population (*n* = 288).

Parameter	ALL (*n* = 288)	Normal group (*n* = 37)	OSA group (*n* = 251)	*P*-value
Male (%)	215 (74.7)	21 (56.8)	194 (77.3)	0.007
Age (years)	46 (35–54)	34 (28–50)	47 (38–55)	0.001
Height (cm)	170 (165–175)	170 (165–177)	171 (165–175)	0.554
Weight (kg)	81 (70–90)	72 (65–80)	83 (73–92)	< 0.001
BMI (kg/m^2^)	27.74 (24.99–30.78)	24.46 (23.55–26.18)	28.34 (25.39–31.21)	< 0.001
NC (cm)	41 (38–43)	38 (36–40)	42 (39–44)	< 0.001
NHR	0.24 (0.22–0.25)	0.22 (0.21–0.23)	0.24 (0.22–0.25)	< 0.001
Headache (%)	88 (30.6)	3 (8.1)	85 (33.9)	0.003
Thirst (%)	209 (72.6)	14 (37.8)	195 (77.7)	< 0.001
Fatigue (%)	185 (64.2)	9 (24.3)	176 (70.1)	< 0.001
Apnea (%)	187 (64.9)	10 (27.0)	177 (70.5)	< 0.001
Snoring (%)	276 (95.8)	31 (83.8)	245 (97.6)	< 0.001
Hypertension (%)	156 (54.2)	4 (10.8)	152 (60.6)	< 0.001
AHI (n/h)	27.4 (11.2–65.4)	2.4 (1.4–4.1)	35.9 (16.4–70.5)	< 0.001
Lowest SpO_2_ (%)	83 (74–87)	87 (81–91)	81 (74–86)	< 0.001
No-Apnea	5 (2–7)	2 (1–3)	5 (3–7)	< 0.001
NOSAS	11 (7–13)	4 (2–7)	11 (8–13)	< 0.001
GOAL	2 (2–3)	1 (1–2)	3 (2–3)	< 0.001
Stop-bang	5 (3–6)	2 (2–3)	5 (4–6)	< 0.001

Continuous and numerical variables reported median (interquartile spacing) and frequency (percentage). BMI, body mass index; NC, the neck circumference; NHR, neck-to-height ratio; OSA, obstructive sleep apnea; AHI, apnea/hypopnea index; SpO_2_, oxygen saturation.

The results showed that at AHI ≥5/h, AHI ≥15/h, AHI ≥30/h, the sensitivity and specificity of GOAL ranged from 88.05 to 94.89% and 56.76 to 29.14%. The sensitivity of GOAL+NC was 70.12–70.80%, and the specificity was 86.49 to 76.16%. The sensitivity and specificity of GOAL+NHR ranged from 73.31 to 81.75% and 83.78 to 70.86%. With the increase of OSA severity, sensitivity increased and specificity decreased. For all levels of OSA, GOAL was more sensitive but less specific than GOAL+NC or GOAL+NHR. Results of PPV, NPV, LR+, and LR− were shown in Table 2.

**TABLE 2:** Performance of the screening tool at different apnea-hypopnea index (AHI) thresholds.

	No-Apnea	NoSAS	STOP-Bang	GOAL	GOAL+NHR	GOAL+NC
**AHI**≥**5/h**						
Sensitivity	81.67 (76.3–86.3)	76.10 (70.3–81.2)	83.67 (78.5–88.0)	88.05 (83.4–91.8)	73.31 (67.4–78.7)	70.12 (64.0–75.7)
Specificity	70.27 (53.0–84.1)	78.38 (61.8–90.2)	70.27 (53.0–84.1)	56.76 (39.5–72.9)	83.78 (68.0–93.8)	86.49 (71.2–95.5)
PPV	94.9 (91.9–96.8)	96.0 (92.8–97.8)	95.0 (92.1–96.9)	93.2 (90.5–95.2)	96.8 (93.6–98.5)	97.2 (93.9–98.8)
NPV	36.1 (28.8–44.1)	32.6 (26.8–39.0)	38.8 (30.9–47.4)	41.2 (31.1–52.0)	31.6 (26.5–37.3)	29.9 (25.3–34.9)
LR+	2.75 (1.67–4.52)	3.52 (1.90–6.53)	2.81 (1.71–4.63)	2.04 (1.40–2.95)	4.52 (2.17–9.44)	5.19 (2.29–11.77)
LR−	0.39 (0.30–0.50)	0.30 (0.23–0.40)	0.23 (0.16–0.33)	0.52 (0.44–0.61)	0.32 (0.25–0.41)	0.35 (0.27–0.43)
**AHI**≥**15/h**						
Sensitivity	88.14 (82.7–92.3)	84.54 (78.7–89.3)	91.75 (87.0–95.2)	91.75 (87.0–95.2)	87.63 (82.2–91.9)	76.29 (69.7–82.1)
Specificity	52.13 (41.6–62.5)	62.77 (52.2–72.5)	54.26 (43.7–64.6)	37.23 (27.5–47.8)	72.34 (62.2–81.1)	79.79 (70.2–87.4)
PPV	79.2 (75.4–82.5)	82.4 (78.2–86.0)	80.5 (76.8–83.8)	75.1 (72.0–78.0)	86.7 (82.4–90.1)	88.6 (83.8–92.1)
NPV	68.1 (58.1–76.6)	66.3 (57.7–73.9)	76.1 (65.8–84.1)	68.6 (56.1–78.9)	73.9 (65.6–80.8)	62.0 (55.4–68.2)
LR+	1.84 (1.48–2.29)	2.27 (1.73–2.97)	2.01 (1.60–2.51)	1.46 (1.24–1.72)	3.17 (2.27–4.41)	3.77 (2.51–5.68)
LR−	0.23 (0.15–0.35)	0.25 (0.17–0.35)	0.15 (0.09–0.25)	0.22 (0.13–0.38)	0.17 (0.12–0.25)	0.30 (0.23–0.39)
**AHI**≥**30/h**						
Sensitivity	90.51 (84.3–94.9)	89.78 (83.4–94.3)	97.81 (93.7–99.5)	94.89 (89.8–97.9)	81.75 (74.3–87.8)	70.80 (62.4–78.3)
Specificity	39.07 (31.2–47.3)	49.67 (41.4–57.9)	42.38 (34.4–50.7)	29.14 (22.0–37.1)	70.86 (62.9–78.0)	76.16 (68.6–82.7)
PPV	57.4 (54.0–60.8)	61.8 (57.8–65.7)	60.6 (57.3–63.9)	54.9 (52.1–57.5)	71.8 (66.2–76.8)	72.9 (66.5–78.5)
NPV	81.9 (72.3–88.8)	84.3 (76.1–90.0)	95.5 (87.3–98.5)	86.3 (74.6–93.1)	81.1 (74.7–86.1)	74.2 (68.6–79.1)
LR+	1.49 (1.29–1.71)	1.78 (1.51–2.11)	1.70 (1.48–1.95)	1.34 (1.20–1.49)	2.81 (2.16–3.64)	2.97 (2.19–4.03)
LR−	0.24 (0.14–0.42)	0.21 (0.12–0.35)	0.05 (0.02–0.16)	0.18 (0.08–0.38)	0.26 (0.18–0.37)	0.38 (0.29–0.51)

AHI, apnea-hypopnea index; GOAL+NHR, GOAL questionnaire combined with neck-to-height ratio; GOAL+NC, GOAL questionnaire combined with neck circumference; PPV, positive predictive value; NPV, negative predictive value; LR+, positive likelihood ratio; LR−, negative likelihood ratio. Data are presented as estimates (95% confidence intervals). Data showed that individuals were classified as at high risk for obstructive sleep (OSA) apnea using the following cut-off points: NoSAS≥ 8 (0–17 points), No-Apnea ≥3 (0–9 points), STOP-Bang ≥3 (0–8 points), GOAL ≥2 (0–4 points).

**FIGURE 1:**
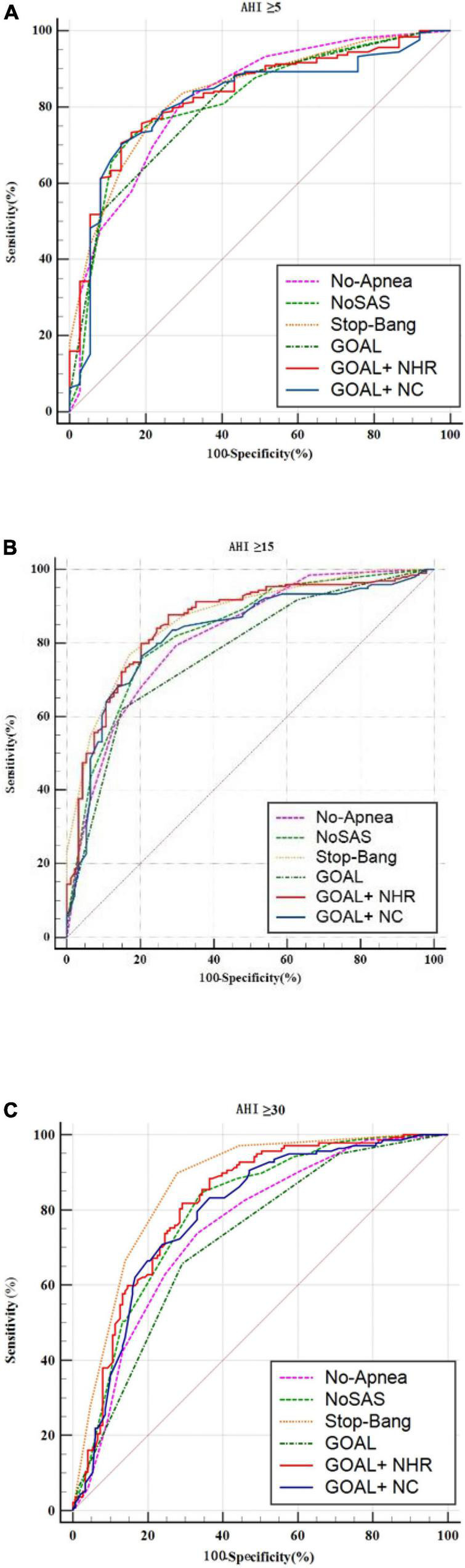
ROC curve of the six screening tools at apnea-hypopnea index (AHI) cut-off points of **(A)** ≥5, **(B)** 15, and **(C)** 30 times/h.

In screening for any OSA, Moderate to severe OSA and severe OSA, the AUC values for No-Apnea was 0.823, 0.819, and 0.750, the AUC values for NoSAS was 0.817, 0.832, and 0.795, the AUC values for GOAL was 0.806, 0.775, and 0.719, the AUC values for GOAL+NC was 0.815, 0.824, and 0.789, the AUC values for GOAL+NHR was 0.831, 0.856, and 0.814, the AUC values for STOP-Bang was 0.837, 0.868, and 0.863. STOP-Bang showed the best results at OSA ≥5, OSA ≥15, and OSA ≥30, followed by GOAL+NHR as shown in Table 3 and Figure 1. See Appendix for pairwise comparison of ROC curves.

**TABLE 3:** Identification ability of screening tools.

	No-Apnea	NoSAS	STOP-Bang	GOAL	GOAL+NHR	GOAL+NC
**AHI ≥5**						
	0.823 (0.774–0.865)	0.817 (0.767–0.860)	0.837 (0.789–0.878)	0.806 (0.756–0.850)	0.831 (0.782–0.872)	0.815 (0.765–0.858)
**AHI ≥15**						
	0.819 (0.769–0.861)	0.832 (0.784–0.873)	0.868 (0.823–0.904)	0.775 (0.723–0.822)	0.856 (0.810–0.895)	0.824 (0.775–0.866)
**AHI ≥30**						
	0.750 (0.696–0.799)	0.795 (0.744–0.840)	0.863 (0.817–0.900)	0.719 (0.663–0.770)	0.814 (0.764–0.857)	0.789 (0.737–0.834)

AHI, apnea-hypopnea index, GOAL+NHR, GOAL questionnaire combined with neck-to-height ratio; GOAL+NC, GOAL questionnaire combined with neck circumference. Data were reported as the area under the curve (AUC) (95% confidence interval). The AUC of the six screening tools were compared with the apnea-hypopnea index (AHI) cut-off points 5, 15, and 30 times/h.

**FIGURE 2:**
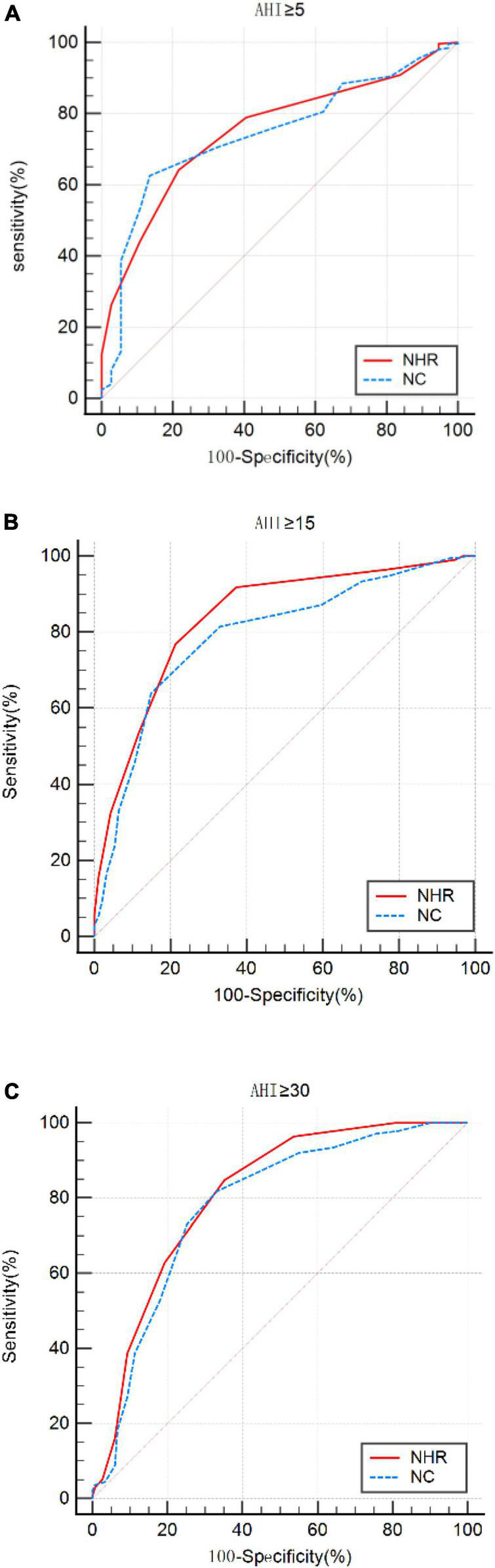
ROC curves for neck-to-height ratio (NHR) and neck circumference (NC) at apnea-hypopnea index (AHI) cut-off points **(A)** ≥5, **(B)** 15, and **(C)** 30 times/h.

Using ROC curves, we evaluated the optimal cut-off points for NHR and NC to detect different levels of OSA. The results showed that at AHI ≥5/h, AHI ≥15/h, and AHI ≥30/h, the NHR cut-off values were all >0.23, and the values for AUC were 0.751, 0.838, and 0.808, respectively, sensitivity of 64.14 to 84.67%, and specificity of 78.38, 78.72, and 64.90%. The results showed that at AHI ≥5/h, AHI ≥15/h, and AHI ≥30/h, the cutoff values for NC were >40, >41, and >40, and the values for AUC were 0.743, 0.790, and 0.779, respectively, the sensitivity ranged from 62.55 to 81.75% and specificity ranged from 86.49 to 66.89%. Results of PPV, NPV, LR+, and LR− were shown in Table 4. The ROC curve was shown in Figure 2.

**TABLE 4:** Screening capacity of neck-to-height ratio (NHR) and neck circumference (NC) at different apnea-hypopnea index (AHI) thresholds.

	Cut-off	AUC	Sensitivity	Specificity	PPV	NPV	LR+	LR−
**AHI ≥5**								
NHR	>0.23	0.751 (0.697–0.800)	64.14 (57.9–70.1)	78.38 (61.8–90.2)	95.3 (91.5–97.4)	24.4 (20.3–29.0)	2.97 (1.60–5.52)	0.46 (0.36–0.58)
NC	>40	0.743 (0.688–0.792)	62.55 (56.2–68.6)	86.49 (71.2–95.5)	96.9 (93.3–98.6)	25.4 (21.7–29.5)	4.63 (2.04–10.52)	0.43 (0.35–0.53)
**AHI ≥15**								
NHR	>0.23	0.838 (0.790–0.878)	76.80 (70.2–82.5)	78.72 (69.1–86.5)	88.2 (83.4–91.7)	62.2 (55.5–68.4)	3.61 (2.43–5.37)	0.29 (0.22–0.39)
NC	>41	0.790 (0.739–0.836)	63.92 (56.7–70.7)	85.11 (76.3–91.6)	89.9 (84.4–93.6)	53.3 (48.2–58.4)	4.29 (2.62–7.04)	0.42 (0.35–0.52)
**AHI ≥30**								
NHR	>0.23	0.808 (0.757–0.851)	84.67 (77.5–90.3)	64.90 (56.7–72.5)	68.6 (63.5–73.3)	82.4 (75.6–87.6)	2.41 (1.92–3.03)	0.24 (0.16–0.36)
NC	>40	0.779 (0.727–0.826)	81.75 (74.3–87.8)	66.89 (58.8–74.3)	69.1 (63.8–74.0)	80.2 (73.6–85.4)	2.47 (1.94–3.14)	0.27 (0.19–0.40)

AHI, apnea-hypopnea index; PPV, positive predictive value; NPV, negative predictive value; LR+, positive likelihood ratio; LR−, negative likelihood ratio. Data are presented as estimates (95% confidence intervals).

## Discussion

The combination of the GOAL questionnaire with NHR and NC (GOAL+NHR and GOAL+NC) was the core of the study, and was then compared with the Stop-Bang questionnaire, No-Apnea score, NOSAS score, and the GOAL questionnaire. As shown in Table 3, AUC values of GOAL+NHR (0.831, 0.856, and 0.814) and GOAL+NC (0.815, 0.824, and 0.789) are significantly higher than those of GOAL (0.806, 0.775, and 0.719). This suggests that the combination of NC and NHR analysis can significantly improve the screening ability of OSA. Similarly, it should not be ignored that the analysis incorporating NHR is superior to the analysis incorporating NC. As shown in Table 4, the AUC values of NHR (0.751, 0.838, and 0.808) were greater than those of NC (0.743, 0.790, and 0.779). In addition, it can be intuitively found that when NHR takes a value greater than 0.23 as a predictor of OSA may be more suitable for the study population. Also, it was demonstrated in the experimental results that GOAL+NHR has better screening ability than NO-Apnea, NOSAS, NHR, and NC, but lower than STOP-Bang (which has the highest AUC). Recent work by Vana et al. (2021) has shown that the NHR has similar discriminatory power to the derived STOP-Bang questionnaire in screening for moderate-to-severe OSA. Ho et al. (2016) shows that NHR can be used as a simple screening tool for OSA in children or adults, which can improve the clinician’s ability to classify children or adults at risk for OSA. Although the above screening methods have achieved good results, the NHR is an indicator that is often rarely considered or seriously ignored in four widely validated OSA screening tools such as STOP-Bang, No-Apnea, NoSAS, and GOAL (Chung et al., 2008; Marti-Soler et al., 2016; Duarte et al., 2018, 2020, 2021, 2022). Therefore, the combination of the NHR to the GOAL questionnaire for OSA screening and analysis is precisely the most significant contribution of this study.

Generally, the specificity, sensitivity, PPV, NPV, LR+, and LR− of screening tools are obtained by 2 × 2 contingency table. Generally, as sensitivity increases, specificity decreases, and vice versa. In our study, the sensitivity of GOAL+NHR and GOAL+NC is lower than Stop-Bang, No-Apnea, NoSAS, and GOAL, but the specificity is higher. Therefore, when our screening tool is employed to determine OSA, it is less likely to result in missed diagnosis. This will not only reduce the number of unnecessary referrals, but also effectively avoid wasting resources. It is important to note that high sensitivity and sufficient specificity of screening tools are generally the desired outcomes of all studies. In this study, although the sensitivity of GOAL+NHR and GOAL+NC at the optimal cut-off value is low (73.31 and 70.12%), they are within the acceptable range (Herschmann et al., 2021), and the fact that they have high specificity (83.78 and 86.49) resulted in robust predictive performance could not be ignored. In addition, both GOAL+NHR and GOAL+NC had higher PPV for any prediction of OSA, which make it therefore much more useful to rule in than to rule out a possible diagnosis of OSA. Furthermore, in the likelihood ratio analysis, when OSA ≥5, the results of GOAL+NHR (positive likelihood ratio 4.52, negative likelihood ratio 0.32) and GOAL+NC (positive likelihood ratio 5.19, negative likelihood ratio 0.35) are superior to the other four screening tools. This indicates that positive screening results of GOAL+NHR and GOAL+NC are more likely to be true positive.

The original GOAL study was developed in a large sample size continuous sleep laboratory (Duarte et al., 2020). All patients were diagnosed with PSG. In the derived cohort (*n* = 3771), the sensitivity and specificity of GOAL for screening OSA ≥5, OSA ≥15, and OSA ≥30 ranged from 83.3 to 94.0% and 62.4 to 38.5%. In the validation cohort (*n* = 3606), GOAL showed similar results. Sensitivity was 83.7 to 94.2% and specificity was 63.4 to 37.7% (cut-off points for screening OSA severity AHI were 5, 15, and 30) (Duarte et al., 2020). The Stop-Bang questionnaire was the first developed and most recognized tool for OSA screening (Chung et al., 2008). Compared with STOP-Bang, GOAL showed similar screening ability and satisfactory predictive performance at OSA ≥5, OSA ≥15, and OSA ≥30. These results were also observed when compared with No-Apnea and NoSAS scores.

During the development of the GOAL questionnaire, seven independent predictors were screened (male gender, age ≥50 years, snoring, BMI ≥30 kg/m^2^, NC ≥40 cm, apnea observed, and hypertension) (Duarte et al., 2020). Models containing 3–7 parameters were calculated for AUC values, respectively. Although the AUC value after including NC was higher than that without NC, the authors finally chose the first 4 clinical parameters as the indicators of the prediction model. Duarte et al. (2020) believed that BMI, rather than NC, was the index to indicate the degree of obesity. However, it is controversial that BMI is used as the only indicator of obesity (Jih et al., 2014). This phenomenon is attributed to the difference in the local distribution of body fat, that is, BMI represents the overall fat distribution, while NC and NHR represent the regional fat distribution. Our views are also consistent with previous studies by Hoffstein and Mateika (1992). Similar studies are also reflected in literature (Vana et al., 2021). Vana et al. (2021) also found that NC and NHR were better indicators of neck obesity than BMI. Similarly, the investigation of Tom et al. (2018) also proved that NC was more correlated with the severity of OSA disease, and the correlation was stronger than BMI. The screening ability of the final GOAL questionnaire, developed by Ricardo LM Duarte, was not affected by NC. Therefore, NC and NHR metrics are employed by us in this work. Therefore, our study combined GOAL questionnaire with NC or NHR to determine the influence of NC or NHR variables on GOAL questionnaire in screening OSA.

## Advantages and limitations

This study focused on OSA predictors and made an exploration. The NHR is usually ignored in predictive tools for OSA (e.g., GOAL questionnaire, STOP-Bang questionnaire, No-Apnea score, NOSAS score), yet we found that the predictive performance of incorporating the NHR in the GOAL questionnaire was superior to incorporating the NC. To our knowledge, this is the first validated design to assess the predictive performance of the GOAL questionnaire combined with the NHR in individuals with OSA. This study can provide an insightful and valuable reference for future research in the development of OSA prediction tools.

Although the metrics required for this study have all been included in every individual who received the PSG or received a home portable sleep test at our institution, there are some limitations to our study. First, some of the data used in the study were retrospective, which is not as good as prospective data. Usually, ideally, all patients should receive the same diagnostic test (e.g., PSG), and this is a limitation of this study. Second, the data in this investigation were mainly from patients in northern China, which did not better reflect the characteristics of other regions (e.g., differences in body fat and height distribution). Last but not least, the small sample size used in this study and the preponderance of severe patients may limit the results of the analysis. Therefore, validity in patients with mild disease needs to be further evaluated.

## Conclusion

Our study shows that the predictive power of the GOAL questionnaire combined with NC and NHR is superior to that of the GOAL questionnaire itself. In addition, GOAL+NHR is superior to GOAL+NC in predicting OSA severity and is superior to both No-Apnea and NoSAS. The use of GOAL questionnaires containing other clinical parameters (such as NHR) is an effective use of medical resources for screening for OSA. Most importantly, we suggest that the inclusion of NHR indicators in OSA screening should be given sufficient attention and consideration.

## Data availability statement

The raw data supporting the conclusions of this article will be made available by the authors, without undue reservation.

## Ethics statement

The studies involving human participants were reviewed and approved by the Ethics Committee of Jinzhou Medical University (LLSC2020008). The patients/participants provided their written informed consent to participate in this study. Written informed consent was obtained from the individual(s) for the publication of any potentially identifiable images or data included in this article.

## Author contributions

YZ conducted the data analysis and wrote this manuscript. CL directed the study. HZ, XY, HY, and LW collected the data for this study. All authors conceived the study.

## Appendix

**TABLE A1:** Pairwise comparison of ROC curves.

	Difference between areas	Standard error	95% Confidence interval	z Statistic	*P*
**(A) AHI ≥5/h.**					
GOAL vs. GOAL+NHR	0.0245	0.0135	−0.00202 to 0.0510	1.811	0.0702
GOAL vs. GOAL+NC	0.00872	0.0158	−0.0222 to 0.0397	0.552	0.5809
GOAL vs. No Apnea	0.0167	0.0300	−0.0421 to 0.0756	0.558	0.5772
GOAL vs. NOSAS	0.0109	0.0290	−0.0460 to 0.0677	0.375	0.7078
GOAL vs. STOP-Bang	0.0310	0.0255	−0.0191 to 0.0810	1.212	0.2256
GOAL+NHR vs. GOAL+NC	0.0158	0.00876	−0.00140 to 0.0329	1.800	0.0718
GOAL+NHR vs. No Apnea	0.00775	0.0252	−0.0416 to 0.0572	0.308	0.7584
GOAL+NHR vs. NOSAS	0.0136	0.0221	−0.0296 to 0.0568	0.618	0.5368
GOAL+NHR vs. STOP-Bang	0.00646	0.0203	−0.0333 to 0.0463	0.318	0.7503
GOAL+NC vs. No Apnea	0.00802	0.0255	−0.0419 to 0.0580	0.315	0.7530
GOAL+NC vs. NOSAS	0.00215	0.0216	−0.0402 to 0.0445	0.0997	0.9206
GOAL+NC vs. STOP-Bang	0.0222	0.0222	−0.0213 to 0.0657	1.002	0.3164
No Apnea vs. NOSAS	0.00587	0.0194	−0.0321 to 0.0438	0.303	0.7619
No Apnea vs. STOP-Bang	0.0142	0.0281	−0.0408 to 0.0692	0.507	0.6125
NOSAS vs. STOP-Bang	0.0201	0.0244	−0.0278 to 0.0679	0.823	0.4108
**(B) AHI ≥15/h.**					
GOAL vs. GOAL+NHR	0.0808	0.0196	0.0423–0.119	4.113	< 0.0001
GOAL vs. GOAL+NC	0.0486	0.0158	0.0176–0.0795	3.075	0.0021
GOAL vs. No Apnea	0.0431	0.0243	−0.00452 to 0.0907	1.774	0.0761
GOAL vs. NOSAS	0.0565	0.0217	0.0140–0.0991	2.604	0.0092
GOAL vs. STOP-Bang	0.0921	0.0213	0.0504–0.134	4.331	< 0.0001
GOAL+NHR vs. GOAL+NC	0.0322	0.0104	0.0118–0.0526	3.092	0.0020
GOAL+NHR vs. No Apnea	0.0377	0.0170	0.00431–0.0710	2.213	0.0269
GOAL+NHR vs. NOSAS	0.0242	0.0151	−0.00529 to 0.0538	1.609	0.1077
GOAL+NHR vs. STOP-Bang	0.0114	0.0189	−0.0257 to 0.0484	0.601	0.5478
GOAL+NC vs. No Apnea	0.00546	0.0178	−0.0295 to 0.0404	0.306	0.7597
GOAL+NC vs. NOSAS	0.00798	0.0147	−0.0207 to 0.0367	0.545	0.5861
GOAL+NC vs. STOP-Bang	0.0436	0.0186	0.00702–0.0801	2.336	0.0195
No Apnea vs. NOSAS	0.0134	0.0187	−0.0233 to 0.0501	0.717	0.4731
No Apnea vs. STOP-Bang	0.0490	0.0212	0.00738–0.0907	2.307	0.0210
NOSAS vs. STOP-Bang	0.0356	0.0187	−0.00102 to 0.0722	1.905	0.0567
**(C) AHI ≥30/h.**					
GOAL vs. GOAL+NHR	0.0945	0.0213	0.0528–0.136	4.445	< 0.0001
GOAL vs. GOAL+NC	0.0697	0.0189	0.0326–0.107	3.681	0.0002
GOAL vs. No Apnea	0.0306	0.0253	−0.0189 to 0.0802	1.211	0.2259
GOAL vs. NOSAS	0.0760	0.0217	0.0334–0.119	3.496	0.0005
GOAL vs. STOP-Bang	0.144	0.0223	0.0999–0.187	6.446	< 0.0001
GOAL+NHR vs. GOAL+NC	0.0248	0.0121	0.00112–0.0485	2.052	0.0401
GOAL+NHR vs. No Apnea	0.0638	0.0182	0.0283–0.0994	3.516	0.0004
GOAL+NHR vs. NOSAS	0.0185	0.0183	−0.0175 to 0.0544	1.007	0.3138
GOAL+NHR vs. STOP-Bang	0.0491	0.0222	0.00557–0.0926	2.211	0.0270
GOAL+NC vs. No Apnea	0.0390	0.0162	0.00733–0.0707	2.413	0.0158
GOAL+NC vs. NOSAS	0.00633	0.0156	−0.0241 to 0.0368	0.407	0.6839
GOAL+NC vs. STOP-Bang	0.0739	0.0210	0.0327–0.115	3.519	0.0004
No Apnea vs. NOSAS	0.0454	0.0218	0.00254–0.0882	2.076	0.0378
No Apnea vs. STOP-Bang	0.113	0.0222	0.0695–0.156	5.094	< 0.0001
NOSAS vs. STOP-Bang	0.0675	0.0207	0.0270–0.108	3.268	0.0011
